# Laparoscopic salvage procedures for adnexal torsion in pediatric and adolescent patients during the COVID-19 pandemic: a retrospective cohort study

**DOI:** 10.1186/s13037-023-00376-7

**Published:** 2023-10-24

**Authors:** Mary Emily Fang, Courtney Crain, Elisabeth Baquet, Jennifer E. Dietrich

**Affiliations:** 1 Department of Obstetrics and Gynecology, Baylor College of Medicine, 1 Baylor Plaza, Houston, TX 77030 USA; 2 Division of Pediatric and Adolescent Gynecology, Texas Children’s Hospital, Houston, TX 77030 USA; 3 Department of Obstetrics and Gynecology, Houston, TX USA; 4 Department of Pediatrics, Baylor College of Medicine, Houston, TX USA

**Keywords:** Adnexal torsion, Laparoscopy, Pediatric and adolescent gynecology, Ovarian torsion

## Abstract

**Background:**

Early management for adnexal torsion increases likelihood of ovarian/tubal salvage. The Coronavirus disease of 2019 (COVID-19) pandemic poses delays from symptom-onset to intervention. The primary objective was to evaluate rates of ovarian salvage and tubal salvage following ovarian torsion and adnexal torsion during the COVID-19 pandemic in a pediatric and adolescent gynecology population.

**Methods:**

This was a retrospective quality improvement cohort study of pediatric and adolescent gynecology patients at a single children’s hospital who underwent laparoscopy for suspected ovarian torsion/adnexal torsion between March 2020 to March 2021. Descriptive statistics and t-tests were utilized.

**Results:**

There were 50 suspected adnexal cases in 47 patients. All underwent laparoscopy, revealing 39 adnexal torsion occurrences in 36 patients and 1 patient with recurrent adnexal torsion three times. All underwent pre-operative COVID-19 testing. Mean age was 13.9 ± 2.6 years for adnexal torsion cohort. Menarche was achieved in 88% (*n* = 44) and 12% (*n* = 6) were pre-menarchal. The primary outcome was ovarian salvage and tubal salvage rates, which were 97.4% (*n* = 38) and 89.7% (*n* = 35), respectively. Secondary outcomes assessed factors contributing to the primary outcome or operative delays. The mean age of menarche was 11.2 years (salvaged) and 12.5 years (non-salvaged) (*p* = 0.04). There were no differences in mean pain duration or mean COVID-19 testing time between groups. Left, right and bilateral adnexal torsion occurred in 42% (*n* = 21), 32% (*n* = 16), and 4% (*n* = 2) respectively. The most common pathologies were paratubal cyst (*n* = 17, 34%) and benign ovarian cyst (*n* = 16, 32%).

**Conclusions:**

Ovarian salvage and tubal salvage rates were 97.4% and 89.7%, respectively during the time frame studied. These salvage rates during the study period are comparable to previous rates in a pre-COVID cohort at our institution. Institutional and departmental quality and safety initiatives likely contributed to this outcome.

## Background

 Ovarian torsion is a gynecologic surgical emergency marked by complete or partial torsion of an ovary on its supporting ligaments [1]. Adnexal torsion occurs when the fallopian tube twists along with the ovary [1, 2]. The pediatric and adolescent gynecology population represents approximately 30% of cases. Laparoscopy is diagnostic and therapeutic, permitting surgical detorsion with adnexal sparing, to attempt to safely preserve ovarian function [3, 4]. Surgical removal of the ovary and/or the fallopian tube may be necessary if malignancy is suspected or the ovary and/or tube are unsalvageable and/or non-viable, posing the risk of infection and long-term risk of loss of ovarian function [1, 3].

Greater elapsed time to presentation and intervention from symptom onset are associated with increased likelihood of oophorectomy at the time of laparoscopy [3–5]. Studies internationally have shown the Coronavirus disease of 2019 (COVID-19) pandemic has delayed presentation to care and time to intervention for preventable and urgent/emergent medical and surgical conditions [6, 7]. Moreover, those who presented after a delay had more severe symptomatology, [8] leading to increased morbidity, compared to the pre-COVID state [8–11]. For conditions requiring surgical management, further delay was posed by pre-operative testing requirements for COVID-19 infection, given risks for aerosolization. Finally, turnaround time for a COVID-19 test result varies based on type of test and loads of detection [12]. Particularly in the early pandemic, there were unique challenges in decision-making for elective procedures in terms of timely access to surgical care; elective procedures were temporarily postponed to contain the spread of COVID-19 and allocate resources and personnel [6, 13]. Long-term consequences of this delay include more advanced presentations and decreased survivorship in conditions that are otherwise manageable and preventable if detected or managed at an earlier stage [6, 13, 14].

Salvage rates for patients with ovarian/adnexal torsion have been used as a quality metric in gynecology [3, 15]. In addition to the high volume of ovarian/adnexal torsion pediatric and adolescent gynecology cases at our institution, over a 10-year period prior to the COVID-19 pandemic, our institution established a high salvage rare superior to the national average (94%) [5]. There is concern that the barriers to management of time-sensitive conditions posed by the COVID-19 pandemic may similarly negatively impact the rates of ovarian and tubal salvage in ovarian/adnexal torsion pediatric and adolescent gynecology patients compared to in the pre-COVID population, [5] though there is a paucity of data investigating this impact. Therefore, we conducted a quality improvement study to explore the impact of the COVID-19 pandemic on rates of ovarian and tubal salvage in the setting of ovarian/adnexal torsion in a pediatric and adolescent gynecology population.

## Methods

**Fig. 1:**
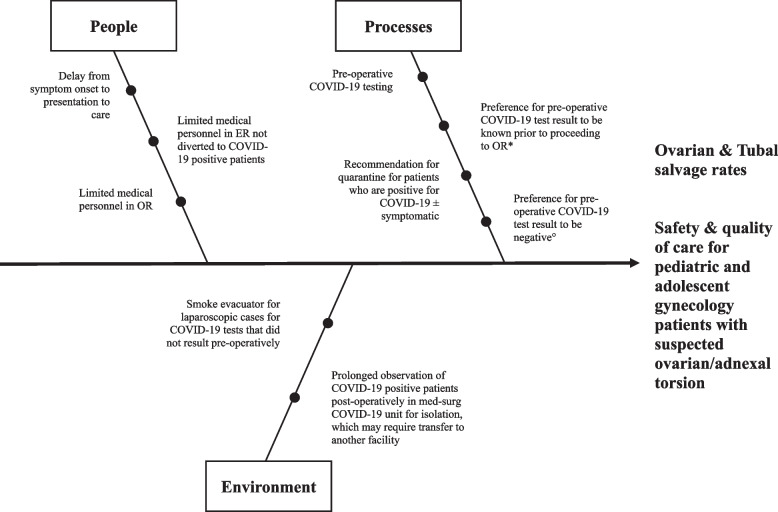
Fishbone diagram to illustrate additional barriers from symptom onset to intervention posed by the COVID-19 pandemic. *Three patients proceeded to the OR prior to pre-operative COVID-19 test resulted. °Two patients had a positive pre-operative COVID-19 test result

This was a retrospective cohort study of patients at a single children’s hospital who underwent laparoscopy for suspected ovarian/adnexal torsion. The institutional review board at Baylor College of Medicine approved this study. The medical records of all children who underwent diagnostic laparoscopy admitted to a single children’s hospital between March 2020 and March 2021 were reviewed retrospectively. Patients who underwent diagnostic laparoscopy for suspected ovarian or adnexal torsion were included in the analysis in regards to baseline demographic characteristics, menarchal status, time to presentation, time from COVID-19 testing to result and relation to operation, procedures performed, intraoperative findings, ovarian and tubal salvage, and pathology findings. The primary outcome was ovarian salvage and tubal salvage rates among the laparoscopically-confirmed ovarian/adnexal cases. Secondary outcomes assessed factors resulting in operative delays or factors contributing to non-salvage (Fig. 1).

Descriptive statistics were utilized. A standardized chart review form was used in the data extraction.

### Statistical analysis

Data were compiled into a computer database. For descriptive analyses, categorical data are expressed as frequencies (percentages), continuous data are expressed as means and standard error of the mean. T tests were utilized to assess differences between groups. A *P* value less than 0.05 determined statistical significance.

## Results

**Fig. 2:**
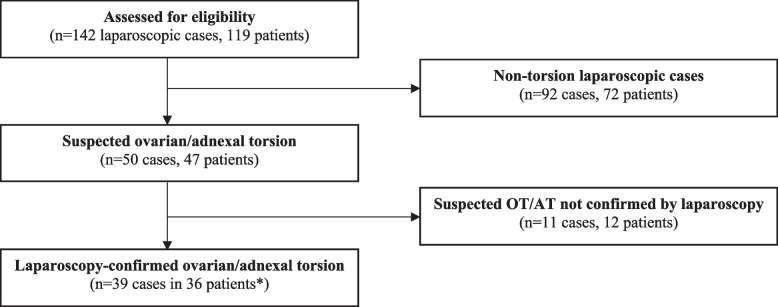
Flow diagram of included patients. *One patient with recurrent torsion 3 times

The chart review identified 142 cases in 119 patients who underwent diagnostic laparoscopy from March 2020 to March 2021. Ninety-two non-torsion laparoscopic cases were excluded from the analysis. There were 50 suspected ovarian/adnexal torsion cases in 47 patients. The mean age was 13.9 ± 2.6 years (range 8–18 years) and 88% (*n* = 44) were post-menarchal, and 12% (*n* = 6) were pre-menarchal. All underwent diagnostic laparoscopy which revealed 39 laparoscopically-confirmed ovarian/adnexal torsion cases in 36 patients with one subject having three episodes of torsion (Fig. 2).

Among the 50 suspected ovarian/adnexal torsion patients, left ovarian/adnexal torsion was confirmed in 42% (*n* = 21/50), right ovarian/adnexal torsion in 32% (*n* = 16/50), bilateral ovarian/adnexal torsion in 4% (*n* = 2/50), and no torsion in 22% of cases (*n* = 11/50). One case involving both adnexa was in a premenarchal patient and the other case was in a menarchal girl who both presented within one day of symptom onset and were found to have paratubal cysts on pathology. The recurrent case was a menarchal patient who experienced 3 recurrences within 3 months involving left side, with spontaneous torsion with no lesion identified on tissue exam.

Among the 39 laparoscopically-confirmed ovarian/adnexal torsion cases, ovarian salvage was accomplished in 97.4% (*n* = 38/39) and tubal salvage in 89.7% (*n* = 35/39), with 1 oophorectomy and 3 salpingectomies in the non-salvaged group, which was the primary outcome of interest. There was one case of autoamputation of the fallopian tube from an old torsion. The mean age of menarche was slightly younger in the salvaged group compared to non-salvaged group (11.2 vs. 12.5 years, *p* = 0.037) (Table 1).

**Table 1:** Comparison of torsion groups

Laparoscopic confirmed torsion cases (*N* = 39)	Adnexal structure Salvaged (*n* = 35)	Adnexal structure Not Salvaged (*n* = 4)	*P* value
Mean Age (years)	13.8 ± 2.6	15 ± 2.35	0.226
Mean age menarche (years)	11.2 ± 1.5	12.5 ± 0.87	0.037
Mean time for COVID-19 test to return (minutes)	158	173	0.418
Mean duration of pain prior to presentation (hours)	61.3	56	0.470

Of the 4 patients with tubal torsion, 3 underwent salpingectomy of the affected tube (Table 2). The one case who underwent oophorectomy was a menarchal girl noted to have the ipsilateral (right) tube encased in adhesion intraoperatively, suggestive of autoamputation, and thus salpingectomy was not pursued

**Table 2:** Characteristics for patients with adnexal structures not salvaged

**Patient**	1	2	3	4
**Age (years)**	13	11	13	13
**Intervention**	Salpingectomy	Oophorectomy	Salpingectomy	Salpingectomy
**Intraoperative findings/Pathology**	Paratubal cyst	Autoamputation of right adnexa with encasement of right tube	Paratubal cyst	Paratubal cyst
**Duration of pain prior to presentation**	10 days	1 day	1 week	2 days
**Time for COVID-19 test to result (minutes)**	81^a^	93	154	367

^a^Pre-operative COVID-19 test did not result prior to proceeding to the operating room due to emergent nature of the case

Secondary outcomes assessed factors that could have contributed to a delay or a risk for non-salvage. Overall, among the 50 suspected ovarian/adnexal torsion cases, presentation was within 24 h of symptom onset in 52% (*n* = 26), after 24 h but within 72 h for 26% (*n* = 13/50), after 72 h but within one week for 16% (*n* = 8/50), and greater than one week for 6% (*n* = 3/50). Among the 39 laparoscopically-confirmed ovarian/adnexal torsion cases, mean pain duration prior to presentation was 61.3 h (salvaged) and 56 h (non-salvaged) (*p* = 0.47) (Table 1). A pre-operative COVID-19 test prior to surgery was collected from all 50 suspected ovarian/adnexal torsion cases prior to surgery, and results were negative in 96% (*n* = 48/50). While all 50 suspected ovarian/adnexal torsion cases received COVID-19 testing, 6% (*n* = 3/50) did not result pre-operatively, with a range of 4–7 medical personnel in the operating room at risk for COVID-19 exposure. The average turnaround time from COVID-19 sample collection to a result was 143+/- 122 min. The mean length of COVID-19 testing was 158 and 173 min for the ovarian/adnexal torsion salvaged and non-salvaged groups respectively (*p* = 0.42) (Table 1). COVID-19 tests resulted within an hour of collection in 18% (*n* = 9/50), within two hours of collection for 42% (*n* = 21/50), within three hours of collection for 22% (*n* = 11/50), within four hours of collection for 10% (*n* = 5/50), and over four hours of collection for 10% (*n* = 5/50).

Regarding the 4 patients who underwent surgical removal (Table 2), all were menarchal, and length of presentation was approximately within 72 h for half (with symptoms for 1 day and 2 days) and after 1 week for the other half (with symptoms for 7 days and 10 days). For the 2 patients who presented to the emergency room (ER) within 72 h of symptom onset, additional delay to the operating room (OR) due to pre-operative COVID-19 testing was 93 min (for the patient who presented within 1 day) and 367 min (for the patient who presented within 2 days). The patient who presented within one day of symptom onset was the case with autoamputation of the right adnexa, with right oophorectomy but no salpingectomy of the encased right tube. For the 2 patients who presented to the ER after 1 week of symptom onset, the patient who presented after 10 days proceeded to the OR urgently; the patient who presented after 7 days had additional delay to the OR due to pre-operative COVID-19 testing that resulted after 154 min.

Regarding the 2 patients whose pre-operative COVID-19 test result was positive, one menarchal patient presented within 2 days of symptom onset was taken to the OR emergently due to severe pain and a large adnexal cyst on ultrasound, despite being incidentally COVID-19 positive and asymptomatic on presentation. Post-operatively, she was transferred to recover in the main campus med-surg COVID-19 unit. The menarchal patient with 3 recurrences had tested positive for COVID-19 on her second recurrence. For the second occurrence, she presented to the ER within 3 days of symptom onset, after her 10-day quarantine from testing positive for COVID-19. For the first and third occurrences, she presented within one day of acute left lower quadrant pain.

Regarding the 3 patients who proceeded to the OR prior to their pre-operative COVID-19 test result, one patient presented after 10-days of symptom onset and proceeded to the OR based on the urgent nature of the case (severe, unresponsive pain and vomiting), and this decision was communicated with the chief of the service. The test result was ultimately negative, resulting after 81 min. The second patient was transferred from an outside hospital, where she tested negative. The third was the patient with 3 episodes of recurrent torsion on her second presentation where she presented after a 10-day quarantine, described above. Her pre-operative COVID-19 test was positive, resulting after 155 min.

Pathology specimens were available for 48 of the 50 suspected ovarian/adnexal torsion cases, with the most common pathology noted to be paratubal cyst (*n* = 17/48, 35.4%), followed by ovarian (*n* = 16/48, 33.3%), tubal/ovarian (*n* = 4/48, 8.3%), and no lesion was identified in 33.3% (*n* = 8/48) (Table 3).

**Table 3:** Intraoperative findings laparoscopic torsion cohort

Operative Cohort^a^ Characteristics	Suspected OT/AT cases (*n* = 50)
Ovarian cyst present	16
Paratubal cyst present	17
Paratubal cyst and ovarian cyst present	4
Not torsed	11
Concurrent findings**	5
Right sided torsion	16
Left sided torsion	21
Bilateral torsion	2

^a^Note that the operative cohort includes all 50 suspected OT/AT cases. Torsion was confirmed in 39 cases

**Concurrent findings include endometriosis, hemorrhagic cyst, follicular cyst, appendix, pyosalpinx

## Discussion

In our retrospective cohort study, ovarian salvage was accomplished in 97.4% patients among those with ovarian torsion and tubal salvage in 89.7% among those with adnexal torsion. The salvage rate of 94% published by Adeyemi-Fowode et al. in 2019 [5] has been reported previously. Therefore, our rate was similar to previously established rates in the literature [5, 15], which was maintained during this study’s timeframe during the COVID-19 pandemic.

Our study analyzed several other secondary outcomes and only one was statistically significant: the mean age of menarche. For those in the salvaged group, the mean age of menarche was slightly younger. This could indicate that the support structures in the pelvis for girls with ovarian salvage were more adult size, resulting in less ability for the utero-ovarian ligament or infundibulopelvic ligaments to twist as tightly as they might in a pre-menarchal girl [16].

There is a paucity of data regarding the effects of the COVID-19 pandemic on ovarian and adnexal salvage. This study addresses this knowledge gap and aims to illustrate how the COVID-19 pandemic impacted our treatment and care of ovarian/adnexal torsion at our institution in the initial stages of the pandemic. Our study fortunately found similar salvage rates to those previously reported in the literature during the time frame studied, and this perhaps points to the fact that there may be more time than expected in order to salvage fallopian tubes and ovaries in an acute adnexal torsion event. While the exact latency from symptom onset to non-viability is not known, Rossi et al [17] had shown there was higher rate of oophorectomy and/or salpingectomy among subjects who presented after 72 h of pain. In our study’s cohort, the mean duration of pain prior to intervention for both groups was under 72 h. The ability to maintain ovarian and tubal salvage rates in the midst of a healthcare crisis is important as this can have lasting effects on the future ovarian function, including fertility, of all those presenting with adnexal torsion during this time [18].

**Fig. 3:**
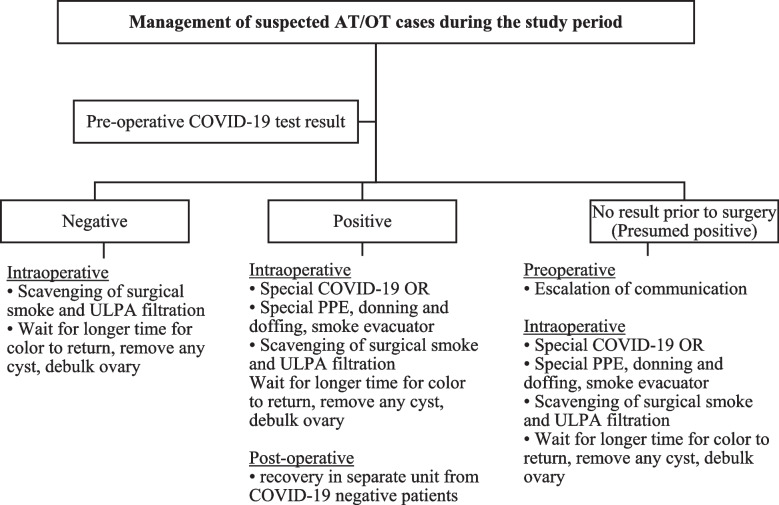
Safety and quality measures in the management of suspected ovarian/adnexal torsion cases during the study period

 The maintenance in the rate of ovarian/adnexal salvage during the study period compared to the established rate prior to the pandemic required active safety and quality interventions at the institutional and pediatric and adolescent gynecology departmental levels. These protocols provided management guidance based on COVID-19 infection status and considered the risks of the urgent/emergent nature of suspected ovarian/adnexal torsion versus risks of COVID positivity or person under investigation (Fig. 3).

In addition to continuing to follow the surgical technique and acumen that contributed to the high salvage rates in the pre-pandemic cohort, additional quality and safety intraoperative measures were implemented to all cases in the study cohort: waiting for a longer period of time for color to return, removing any cysts that may have caused the pedicle to twist, and debulking the ovary to reduce mass effect in order to eliminate any compartment syndrome due to ovarian edema and absent ovarian flow. These active measures were in part to address the delays in presentation and intervention posed by COVID-19, and to minimize the risk of re-torsion, which would risk exposing patients to the same COVID-related delays. Standard operating room safety measures were consistent with recommendations from multiple societies [19, 20] and from statements by authors for the *Journal of Minimally Invasive Gynecology* [21–25] (Fig. 3). Patients who proceeded to the OR prior to pre-operative test results (COVID-19 unknown) were treated as persons under investigation (PUI), and the same safety measures to mitigate the risk of presumed COVID-19 were taken as those who were confirmed positive for COVID-19 (Fig. 3). In our cohort, all 3 patients who proceeded to the OR prior to pre-operative COVID-19 test result had acute presentations requiring urgent/emergent surgical intervention. For these patients, the risk of time-sensitive risk of irreversible damage to the ovary/adnexa took priority over the risks of COVID-19, which were subsequently preemptively addressed by treating these patients with the same safety protocols for COVID-19 positive patients. OR safety measures were followed to minimize COVID-19 exposures to staff and other patients preoperatively, intraoperatively and post operatively. These measures were focused on education, special equipment for gas and smoke scavenge, OR designation, and safety checklist implementation (Fig. 3).

The retrospective nature of this single-center study limits the generalizability of the results to other pediatric and adolescent gynecology populations at other institutions. Further, the delay in COVID-19 testing results was dependent on a number of factors. The time to result may not be similar at every institution. Our data shows that the ovarian and tubal salvage rates were similar during the pandemic compared to prior to the pandemic at our institution alone; however, this finding may not be generalizable to all institutions. In addition, historical salvage rates reported at our institution were recorded as part of a qualitative initiative, and time from presentation to the operating room was not recorded. The time differences between the COVID-19 torsion cohort and historic torsion salvage group therefore could not be compared.

## Conclusion

In conclusion, the rates of ovarian and tubal salvage during the COVID-19 pandemic at our institution were similar to that previously established in the literature. There were no statistically significant differences in the duration of pain or COVID-19 test result time between salvaged and non-salvaged groups. A major clinical implication of reporting these salvage rates is the opportunity for preserved ovarian function in a pediatric and adolescent gynecology population with ovarian/adnexal torsion, which stresses the importance of reviewing outcomes from this retrospective quality improvement study. Additionally, we describe active safety and quality measures preoperatively, intraoperatively, and postoperatively at the institutional and departmental levels that contributed to the maintenance in our ovarian/tubal salvage rates, despite the barriers posed by the COVID-19 pandemic during the study period.

## Data Availability

The datasets used and/or analyzed during the current study are available from the corresponding author on reasonable request.

## Abbreviations

ER: Emergency room
OR: Operating room
ULPA: Ultra-Low Penetration Air filter
